# *De novo* whole-genome assembly in an interspecific hybrid table grape, ‘Shine Muscat’

**DOI:** 10.1093/dnares/dsac040

**Published:** 2022-11-07

**Authors:** Kenta Shirasawa, Hideki Hirakawa, Akifumi Azuma, Fumiya Taniguchi, Toshiya Yamamoto, Akihiko Sato, Andrea Ghelfi, Sachiko N Isobe

**Affiliations:** Kazusa DNA Research Institute, Kisarazu, Chiba, Japan; Kazusa DNA Research Institute, Kisarazu, Chiba, Japan; Institute of Fruit Tree and Tea Science, National Agriculture and Food Research Organization (NARO), Tsukuba, Ibaraki, Japan; Institute of Fruit Tree and Tea Science, National Agriculture and Food Research Organization (NARO), Tsukuba, Ibaraki, Japan; Institute of Fruit Tree and Tea Science, National Agriculture and Food Research Organization (NARO), Tsukuba, Ibaraki, Japan; Institute of Fruit Tree and Tea Science, National Agriculture and Food Research Organization (NARO), Tsukuba, Ibaraki, Japan; Kazusa DNA Research Institute, Kisarazu, Chiba, Japan; Kazusa DNA Research Institute, Kisarazu, Chiba, Japan

**Keywords:** *Vitis labruscana* × *V. vinifera*, Shine Muscat, Genome sequence, phased, unphased

## Abstract

The first genome sequence of an interspecific grape hybrid (*Vitis labruscana* × *Vitis vinifera*), ‘Shine Muscat’, an elite table grape cultivar bred in Japan, is presented. The resultant genome assemblies included two types of sequences: a haplotype-phased sequence of the highly heterozygous genomes and an unphased sequence representing a ‘pseudo-haploid’ genome. The unphased sequences, assembled to the chromosome level with Hi-C reads, spanned 488.97 Mb in length, 99.1% of the estimated genome size, with 4,595 scaffold sequences and a 23.9-Mb N50 length. The phased sequences had 15,650 scaffolds spanning 1.0 Gb and a 4.2-Mb N50 length. 32,827 high-confidence genes were predicted on the unphased genomes. Clustering analysis of the ‘Shine Muscat’ gene sequences with three other *Vitis* species and Arabidopsis indicated that 11,279 orthologous gene clusters were common to *Vitis* spp. and Arabidopsis, 4,385 were *Vitis* specific, and 234 were ‘Shine Muscat’ specific. Whole-genome resequencing was also performed for the parental lines of ‘Shine Muscat’, Akitsu-21 and ‘Hakunan’, and parental-specific copy number variations were identified. The obtained genome resources provide new insights that could assist in cultivation and breeding strategies to produce high-quality table grapes.

## 1. Introduction

Grape (*Vitis* spp., 2*n* = 2*x* = 38) is one of the most widely cultivated and valuable horticultural crops in the world. World grape production in 2019 was 78,034 kt,^1^ with the largest producers being China (14,843 kt), Italy (8,222 kt), Spain (6,818 kt), France (5,884 kt), the USA (5,389 kt), and Turkey (4,209 kt). This production is mainly for wine, but a considerable portion is also for table fruit use. The most common species used for the production of wine and table grapes around the world is the European grape (*V. vinifera* L.).^2,3^*Vitis vinifera* is adapted to a warm, dry climate during its growing season, and countries with a high production of this species have areas with such a climate. However, berries of this species are highly susceptible to fungal diseases under humid conditions.^4^ For example, the failures of early colonists to establish *V. vinifera* on the east coast of North America resulted from a lack of resistance to native diseases, soil pests, and low winter temperatures in the northernmost areas.^5^ To overcome this difficulty, seedlings or selections from wild American native grape species that survived under North American conditions were collected, and many breeders attempted to improve the American native species through hybridization among *V. vinifera* and American native species in the latter half of the 19th century.^6^ The species most often used as a cross parent was *V. labrusca* (the fox grape), which provided disease and cold resistance as well as a distinctive flavor.^5^

*Vitis labruscana* L.H. Bailey is defined as a subgroup of grapes that originated from hybridization of *V. labrusca* with other species, most commonly *V. vinifera*,^7^ and more than 1,500 *V. labruscana* varieties, such as ‘Campbell Early’, ‘Catawba’, ‘Concord’, ‘Delaware’, and ‘Niagara’, have been developed.^4,6^ Japan has a humid climate throughout its growing season and grapes grown in Japan are susceptible to attack from fungal diseases during periods of rainfall. This climate limitation has hampered the development of *V. vinifera* production in Japan. Consequently, Japanese grape breeders have attempted to develop new cultivars through interspecific hybridizations to combine crisp flesh and favorable flavor traits derived from *V. vinifera* grapes, with ease of cultivation (mainly disease and berry cracking resistance) from *V. labruscana* grapes.

‘Shine Muscat’ is a promising cultivar in Japan, bred at the Institute of Fruit Tree and Tea Science, National Agriculture and Food Research Organization (NARO), and derived from a cross between Akitsu-21 and ‘Hakunan’. An offspring grape with crisp flesh, Akitsu-21 (*V. labruscana* × *V. vinifera*) was obtained by crossing ‘Steuben’ (*V. labruscana*) with ‘Muscat of Alexandria’ (*V. vinifera*). Akitsu-21 was then crossed with ‘Hakunan’ (*V. vinifera*) to develop ‘Shine Muscat’ (*V. labruscana* × *V. vinifera*).^8–10^ Because of the favorable fruit eating quality of this grape when produced under Japanese climatic and environmental conditions, together with its sophisticated cultivation system, the cultivation area of ‘Shine Muscat’ rapidly increased after its release by NARO in 2006 and reached 1,840 ha in 2019.^11^ The ‘Shine Muscat’ grape cultivar has large yellow-green berries, crisp flesh texture, muscat flavor, high soluble solids concentration and low acidity. Berries can be eaten with the skin. It is moderately tolerant to downy mildew and ripe rot, but sensitive to anthracnose.^8,12,13^ The shelf life is longer than that of ‘Kyoho’ (*V. labruscana* × *V-+. vinifera*, 2*n* = 4*x* = 76), the current leading cultivar in Japan, and its cold hardiness is comparable with that of ‘Kyoho’.

Owing to the importance of grapes to the food industry, whole genome sequencing in grapevine (*V. vinifera*) has been conducted for approximately fifteen years, with the first draft genome being reported in 2007.^14^ This 2007 draft genome report was the first such report in bearing fruit crops, the second in tree species, and the fourth in higher plant species for which whole-genome sequencing was reported.^15^ The original genome project used a *V. vinifera* cultivar, ‘Pinot Noir’. Since the original genome publication, the PN40024 genome sequence data (12X) have been updated, and the last version was released in 2017.^16^ In addition, *de novo* genome sequence assemblies have been reported in many wine cultivars of *V. vinifera*, such as ‘Cabernet Sauvignon’,^17^ ‘Carménère’,^18^ ‘Chardonnay’,^19,20^ and ‘Zinfandel’.^21^ As of June 2022, the genomes of 12 *V. vinifera* varieties had been published on grapegenomics.com (http://www.grapegenomics.com/). In parallel with the availability of this information, the *Vitis* pan-genome was released, for which whole-genome sequences of as many as 472 accessions of 48 species were sequenced to find structure variations as well as sequence variations across the *Vitis* diversity panel.^22^ The genomes of two wild grapevine species, *V. vinifera* subspp. *sylvestris* and *V. riparia*, were also sequenced by Badouin et al.^23^ and Patel et al.,^24^ respectively. However, little genome information for *V. labruscana* × *V. vinifera* hybrids is available at present.

Genomics has the potential to enhance cultivation and breeding strategies toward the production of high-quality fruits, vegetables, and flowers with attractive consumer-friendly phenotypes. With the advanced technologies and methods available, it has become possible to achieve sequencing analysis of the complex interspecific hybrid genomes often observed in elite cultivars of horticultural crops such as the ‘Shine Muscat’ table grape. In this study, we determined the genome sequences of ‘Shine Muscat’, an interspecific grape hybrid (*V. labruscana* × *V. vinifera*). In addition, we used full-length transcriptome sequencing to gain insights into a gene set associated with elite table grape characteristics.

## 2. Materials and methods

### 2.1. Plant materials

For genome assembly, DNA was extracted from young leaves (expanded to ~6 cm) collected from a vine of ‘Shine Muscat’ (*V. labruscana* × *V. vinifera*) growing in a vineyard at the Institute of Fruit Tree and Tea Science, NARO in Hiroshima, Japan with the use of a Genomic DNA Extraction Column (Favorgen Biotech Corp., Ping-Tung, Taiwan). For transcriptome analysis, total RNA was extracted from young leaves, tendrils, and flower clusters before flowering, and from the skins, flesh, and seeds of mature berries at harvest according to the protocol described by Reid et al.^25^ A total of 220 F_1_ individuals derived from crosses between the ‘Shine Muscat’ parental lines, Akitsu-21 and ‘Hakunan’, were used for linkage map construction.

### 2.2. Genome sequence analysis

Paired-end (PE) libraries (insert sizes of 450–470 and 700–800 bp) and mate-pair (MP) libraries (insert sizes of 2–4, 5–7, and 8–10 kb) were constructed with a KAPA Hyper Prep Kit (Kapa Biosystems, Roche) and Nextera Mate Pair Library Preparation Kit, respectively. A 10× Genomics Chromium library was also prepared. The libraries were sequenced on a HiSeq 2500 system (Illumina, San Diego, CA) in PE 250-bp mode for the PE library, which had insert sizes of 450–470 bp, and on a NovaSeq 6000 system (Illumina) in PE 150-bp mode for the remaining five libraries (Supplementary Table S1). A Hi-C library was constructed from the young leaves as described by Lieberman-Aiden et al. (2009),^26^ using a Proximo Hi-C Plant Kit (Phase Genomics, Seattle, WA), and sequenced by a Hiseq X system.

### 2.3. Genome sequence assembly

The PE reads were used for genome size estimation based on k-mer frequency with Jellyfish. GenomeScope 1.0 was also used to estimate the size and heterozygosity in the genomes. PE, MP, and 10× Genomics sequences were assembled using DeNovoMAGIC3 to generate both unphased and phased genome sequences. Small sequences not integrated into the assemblies were gathered as unplaced sequences. The Hi-C reads were mapped onto the unphased scaffold sequences by BWA. The read pairs with an unmapped mate were removed by SAMtools using -F 12 filtering. Then, chromosome-scale scaffolding was performed by using a Proximo Hi-C genome scaffolding platform (Phase Genomics), and Juicebox was used to correct scaffolding errors.

The phasing accuracy in the phased genome sequences was investigated by mapping whole genome shotgun sequences of parents of ‘Shine Muscat’, Akitsu 21 and ‘Hakunan’ onto the phased genome sequences using Bowtie2. A variant call was performed by bcftools 0.1.19 mpileup in SAMtools, and filtering of variants by quality was performed using vcftools. The identified variants were filtered out with the following parameters: QUAL = 999, DP ≥ 20, GQ ≥ 20 and max-missing = 1. The donor of each scaffold was estimated based on the percentages of allele types of parental genomes under the following conditions. 1) The donor was considered to be Akitsu 21 if the allele ratios of the variants on the scaffold were hetero ≥ 70% on the Akitsu 21 genome, or, alternatively, (Alt) homo ≥ 70% on the ‘Hakunan’ genome. 2) The donor was considered to be ‘Hakunan’ if the allele ratios of the variants on the scaffold were Alt homo ≥ 70% on the Akitsu 21 genome and reference (Ref) Homo ≥ 70% on the ‘Hakunan’ genome.

Variants (SNPs and Indels) segregating in the F_1_ population (Akitsu21 × ‘Hakunan’) with the dd-RAD-Seq reads were then searched against the scaffolds created with Hi-C. Library construction was performed according to Shirasawa et al.^27^ and the dd-RAD-Seq sequences were obtained by Illumina HiSeq 4000. A variant call was performed by bcftools 0.1.19 mpileup in SAMtools, and filtering of variants by quality was performed using vcftools. A linkage map was constructed by using Lep-MAP3. The positions of variants identified on the linkage map and the Hi-C scaffolds were compared to confirm the adequacy of Hi-C scaffolding.

The integrity of the assemblies was also verified using the Benchmarking Universal Single-Copy Orthologs (BUSCO) method. Repetitive sequences in the assembled genome were searched by RepeatMasker for known repetitive sequences registered in Repbase and *de novo* repetitive sequences defined by RepeatModeler. Comparative analysis of the genome structures was performed using D-GENEIS or Nucmer.

### 2.4. Transcriptome analysis and gene prediction

Total RNAs extracted from six tissues of ‘Shine Muscat’ were mixed to prepare an Iso-Seq library in accordance with the manufacturer’s protocol (Pacific Biosciences, Menlo Park, CA). The library was sequenced with single molecule real-time sequencing technology on a Sequel system. The obtained reads were clustered using the Iso-Seq 3 pipeline implemented in SMRT Link, mapped on the unphased sequence of the ‘Shine Muscat’ genome with Minmap2, and collapsed to obtain nonredundant isoform sequences using a module in Cupcake ToFU. Open reading frame (ORF) sequences on the collapsed sequences were identified by using ANGEL. Redundant sequences were then removed by the CD-HIT program, and nonredundant complete confidence (cc) sequences were mapped onto the assembled genome sequences by GMAP.

Meanwhile, empirical gene prediction was performed by BRAKER 2 using the 17 transcript sequences from the Sequence Read Archive (SRA) data derived from *V. vinifera* ‘Muscat Humburg’, *V. labrusca* and *V. labrusca* × *V. vinifera* cultivar ‘Labruscan’ (Supplementary Table S3). To classify the predicted gene sequences based on the evidence level, similarity searches were performed against the NCBI NR protein database (http://www.ncbi.nlm.nih.gov) and UniProtKB (https://www.uniprot.org) using DIAMOND with a mapped length of ≥95% and ≤105%, and an *E*-value ≤1E−100. BLASTP searches were also performed for the gene sequences of *Vitis vinifera* (12X)^16^ and *Arabidopsis thaliana* (Araport 11)^28^ with *E*-values ≤1E−80. A domain search was performed by HAMMER (http://hmmer.org/) with an *E*-value ≤1E−20. Transcript per million (TPM) values were calculated by Salmon with the RNA-Seq reads listed in Supplementary Table S3. The high confidence (HC) gene sequences were selected under the following conditions: TPM value >0.5, identified protein domain sequences, gene sequence hits in the UniProtKB or NR protein database, or *V. vinifera* (12X) genes. Transposon elements (TEs) were classified based on the results of similarity searches against UniProtKB. The gene sequences not classified as HC or TE were classified as LC (low confidence). The gene sets predicted by ANGEL and BRAKER 2, respectively, were then merged. The genes that showed longer ORF sequences were selected when identical genes were predicted by both methods.

Functional gene annotation was also performed by using a modified version of Hayai annotation called ZenAnnotation (https://github.com/aghelfi/ZenAnnotation), in which OrthoDB sequences (https://www.orthodb.org/) were incorporated in order to allow contaminant detection. The predicted gene sequences were clustered by the CD-HIT program with those of *A. thaliana* (Araport11), *V. vinifera* (12X), *V. vinifera* subspp. *sylvestris* (Sylvestris_C1-2_haplotigs_pseudomolecules) and *V. riparia* (EGFV_Vit.rip_1.0).

### 2.5. Estimation of progenitors of phased sequences and copy number variation between ‘Shine Muscat’ and its parental genomes

The parental genomes of ‘Shine muscat’, Akitsu-21 and ‘Hakunan’, were re-sequenced to identify variants between these genomes and the ‘Shine Muscat’ genome. DNAs were extracted from young leaves. PE libraries (insert sizes of 450–470 bp) were constructed with the KAPA Hyper Prep Kit. Sequencing was performed by using the HiSeq X system (Illumina) in PE 150-bp mode. The obtained reads were mapped onto the assembled ‘Shine Muscat’ genome using Bowtie 2. A variant call was performed by bcftools 0.1.19 mpileup in SAMtools, and filtering of variants by quality was performed using vcftools. Donor parents of phased sequences were estimated based on the percentages of allele types detected on each parental genome sequence. Copy number variations (CNVs) were identified for Akitsu-21 and ‘Hakunan’ against ‘Shine Muscat’ using CNV-Seq with a 1 Mb window.

## 3. Results and discussion

### 3.1. Sequencing and assembly

DNA from the leaves of ‘Shine Muscat’ was sequenced. In total, 129.7 and 105.4 Gb sequence data were obtained from the Illumina PE and MP libraries, respectively, as well as 37.0 Gb from a 10× Genomics Chromium library (Supplementary Table S1). The distribution of distinct k-mers (*k* = 17) showed two peaks at multiplicities of 111 and 225, indicating heterozygous and homozygous regions, respectively (Supplementary Fig. S1). This suggested that the heterozygosity of the ‘Shine Muscat’ genome was high, indicative of an interspecific hybrid. The haploid (n) size of the ‘Shine Muscat’ genome was estimated to be 493.0 Mb and the diploid (2*n*) size was estimated to be 999.2 Mb.

Whole-genome sequencing was also performed for the parents of ‘Shine Muscat’, Akitsu-21 and ‘Hakunan’. The estimated genome sizes of Akitsu-21 and Hakunan by Jellyfish were 551.6 and 551.1 Mb, respectively (Supplementary Fig. S1). Meanwhile, genome size estimation performed by GenomeScope 1.0 resulted in genome sizes of 374.1, 447.2, and 455.1 Mb for ‘Shine Muscat’, Akitsu-21 and ‘Hakunan’, respectively (Supplementary Fig. S2). According to Lodhi and Reisch (1995), the DNA content varied from 0.98 to 1.05 pg/2C in *V. labrusca* and from 0.86 to 1.00 pg/2C in *V. vinifera*.^29^ The estimated genome size by Jellyfish in ‘Shine Muscat’ agreed with the previous report, but the genome sizes of Akitsu-21 and ‘Hakunan’ were larger than those reported previously. In contrast, the estimated genome sizes in ‘Shine Muscat’ by GenomeScope were smaller than in the previous studies, and the estimated genome sizes of Akitsu-21 and ‘Hakunan’ agreed with those in the previous studies. In this study, we used the results for Jellyfish to estimate the genome size for ‘Shine Muscat’, which was estimated as 493.0 Mb.

Heterozygosity in the genome estimated by GenomeScope was 1.79%, 1.85%, and 1.32% in ‘Shine Muscat’, Akitsu 21 and ‘Hakunan’, respectively (Supplementary Fig. S2). Akitsu-21 (*V. labruscana* × *V. vinifera*) was obtained by crossing ‘Steuben’ (*V. labruscana*) with ‘Muscat of Alexandria’ (*V. vinifera*), while ‘Hakunan’ was derived from a cross between ‘Katta Kurgan’ (*V. vinifera*) and ‘Kaiji’ (*V. vinifera)*.^10^ The estimated heterozygosity of ‘Shine Muscat’ was intermediate between that of the parents, and closer to the heterozygosity of ‘Akitsu-21’. The highest heterozygosity in Akitsu 21 and the lowest heterozygosity in ‘Hakunan’ appropriately reflected the degree of polymorphism inferred from the pedigree.

The sequence reads from the PE, MP, and 10x Genomics Chromium libraries were assembled to obtain phased and unphased genome sequences, which were designated VSMph_r1.0 and VSMuph_r1.0, respectively (Supplementary Table S4). The phased sequences, VSMph_r1.0, consisted of 15,650 scaffold sequences covering 1,004.7 Mb with an N50 of 4.2 Mb. The unphased sequences, VSMuph_r1.0, were created from the phased sequences. The number of unphased sequences was 8,696, and the total length was 490.1 Mb, almost half the length of the phased sequences. A total of 361,173 sequences covering 175.9 Mb were classified as unplaced sequences during the process of unphasing.

The VSMuph_r1.0 sequences were then scaffolded with the Hi-C sequences. The total number of resultant sequences (hereinafter Hi-C scaffolds) was 8,294, or 402 less than that the total number of VSMuph_r1.0 sequences. The N50 length was 23.9 Mb, or 1.8 times longer than that of VSMuph_r1.0. The results suggest that the major scaffold sequences constituting the chromosomes were scaffolded with the Hi-C reads, whereas most of the fragmented sequences with short lengths in VSMuph_r1.0 were not placed on the chromosome-level scaffolds.

An F_1_ linkage map was also constructed using the variants identified on the Hi-C scaffolds in order to confirm the adequacy of the scaffolding result with the Hi-C reads. The dd-RAD-Seq sequences of the 220 F_1_ individuals were mapped onto the 8,294 Hi-C scaffolds, and a total of 17,510 variants showing segregation in the population were used for the linkage map construction. The 17,510 variants were then grouped with respect to their physical positions on the Hi-C scaffolds. Parental-specific maps and an integrated map were constructed, and a total of 17,031 variants were mapped onto the 19 longest sequences in the Hi-C scaffolds (Supplementary Table S5). Consistent correlation was observed between the linkage and physical positions in each parental specific and integrated map, suggesting that the 19 longest Hi-C scaffolds reflected the 19 chromosomes of the ‘Shine Muscat’ genome (Fig. 2, Supplementary Fig. S3).

The scaffold sequences having a length of ≥500 bp were then designated as VSMuph_r2.0, and the 19 longest scaffolds were given chromosome numbers corresponding to that of the *V. vinifera* genome (12X)^16^ as pseudomolecules. The number of sequences and total length of VSMuph_r2.0 were 4,595 and 489.0 Mb, respectively (Table 1, Supplementary Table S4). The total length of the 19 pseudomolecules was 462.9 Mb, which accounted for 94.4% of the total length of VSMuph_r2.0.

**Table 1.** Statistics of the assembled ‘Shine Muscat’ genome and gene sequences

Sequence name	Unphased genome		Phased genome	Gene
	VSMuph_r2.0.	VSMuph_r2.0.	VSMph_r1.0	VSMuph_r2.0.1
	All sequences	chr01-19		
Number of sequences	4,595	19	15,650	32,827
Total length (bp)	488,971,633	462,883,708	1,004,717,561	41,873,115
N50 length (bp)	23,937,529	23,937,529	4,205,710	1,276
Max length (bp)	35,651,361	35,651,361	46,730,467	17,916
Gaps (%)	3.57	3.48	3.91	0.01
GC (%)	35.3	35.2	35.2	45.0
*BUSCOs (%) v3, obd10*				
Complete	95.3	95.0	96.3	88.9
Complete single copy	93.6	93.4	21.2	86.1
Complete duplicated	1.7	1.5	75.1	2.8
Fragmented	1.4	1.3	1.0	5.6
Missing	3.3	3.7	2.8	5.5

Next, the assembly quality of VSMuph_r2.0 was investigated by mapping the sequences onto 1,375 BUSCOs (Table 1). The results demonstrated that the number of complete BUSCOs was 1,310 (95.3%), including 1,287 (93.6%) single-copy genes and 23 (1.7%) duplicated genes. With respect to the phased sequences, VSMph_r1.0 contained 96.3% complete BUSCOs (21.2% single copy and 75.1% duplicated). The higher ratios of complete BUSCOs in the phased and unphased genomes suggested the high quality of each assembly. The percentage of duplicated complete BUSCOs in the unphased genome was low (1.7%), while that in the phased genome was high (75.1%), suggesting that the phased genome, VSMph_r1.0, reflected the two haploid genome sequences in ‘Shine Muscat’. The sequence contiguity and quality, which were supported by the N50 length and BUSCO analysis, respectively, compared favorably with those of the reported grapevine genomes.^14–18,20,21^

The total length of repetitive sequences in VSMuph_r2.0 (all sequences) was 234.7 Mb, and these sequences occupied 48.0% of the ‘Shine Muscat’ genome (Supplementary Table S6). Of the 234.7 Mb repeat sequences, sequences totaling 203.5 Mb in length were known repeats, occupying 41.6% of the assembled genome. The ratio of known repeat sequences on the genome was almost the same as reported on the 12X genome (41.1%).^14^ LTR elements were most frequently observed, followed by LINEs.

To determine the degree to which the two haplotype-derived sequences were collapsed in the unphased assembly, the unphased genome sequences (VSMph_r1.0) were compared with those in the 19 pseudomolecules in the VSMuph_r2.0 by using D-Genies (Supplementary Fig. S4). In most of the regions, the sequences in VSMph_r1.0 were aligned on the VSMuph_r2.0 while overlapping. Therefore, we considered that the two haplotype-derived sequences were appropriately collapsed to create the unphased sequences. Eight VSMph_r1.0 sequences were overlapped on half of chr5 of VSMuph_r2.0. The excessive duplicate sequences in this region were probably generated due to miss-assembly of VSMph_r1.0.

In addition, the phasing accuracy was validated by mapping whole genome shotgun sequences of parents of ‘Shine Muscat’, Akitsu 21 and ‘Hakunan’, onto VSMph_r1.0 sequences (Supplementary Fig. S5). A total of 514,179 variants with a total length of 911 Mb were identified on the 2,403 sequences (Supplementary Table S7A). No reference homo (Ref homo) alleles were identified on the Akitsu 21 genome, while Ref homo alleles were most frequently observed on the ‘Hakunan’ genome (Supplementary Table S7B). The genome sequences derived from *V. labruscana* were the cause of the absence of Ref homo in Akitsu 21. The possible donor parents on the phased sequences were estimated based on the percentages of allele types of parental genomes, as shown in Table 2. A total of 743 (353.7 Mb, 35.2% to total length of VSMph_r1.0) and 970 (402.0 Mb, 40.0%) sequences were inferred to have their donors Akitsu 21 and ‘Hakunan’, respectively. Six hundred and ninety sequences with a total length of 155.3 Mb were not determined their donors, considering that these sequences were present in both the Akitsu 21 and ‘Hakunan’ genomes. Because 75.2% of sequences were determined their donors, we considered that phasing was adequately performed in the construction of VSMph_r1.0.

**Table 2.** Numbers and total length of scaffolds that determined the possible donor parent

Donor^a^	Number of scaffolds	Total length	
		bp	% to VSMph_r1.0
Akitsu high confidence	183	113,484,905	11.3
Akitsu low confidence	560	240,237,600	23.9
Akitsu total	743	353,722,505	35.2
Hakunan high confidence	516	185,520,858	18.5
Hakunan low confidence	454	216,512,960	21.5
Hakunan total	970	402,033,818	40
Undetermined	690	155,251,616	15.5
Not investigated^b^	13,247	93,709,622	9.3

^a^The donor of each scaffold was estimated based on the percentages of allele types of parental genomes under the following conditions:

Donor = Akitsu 21, Hetero ≥ 70% on the Akitsu 21 genome, Alt Homo ≥ 70% on the ‘Hakunan’ genome.

Donor = ‘Hakunan’, Alt Homo ≥ 70% on the Akitsu 21 genome, Ref Homo ≥ 70% on the ‘Hakunan’ genome.

High confidence: The donor was estimated by both parental genomes, and the results matched.

Low confidence: Either of the parental genomes determined the donor, while another parental genome gave an ‘undetermined’ result.

^b^Sequences excluded from investigation because no variants were identified.

### 3.2. Comparisons with other *Vitis* genomes at the genome sequence level

The genome sequences of the 19 pseudomolecules in the VSMup_r2.0 genome were compared with those in *V. vinifera*^16^ (12X), *V. vinifera* subspp. *sylvestris* (Sylvestris C1-2)^23^ and *V. riparia* (EGFV_Vit.rip_1.0).^24^ High sequence homology was observed between the VSMup_r2.0 and the three compared *Vitis* genomes in most regions (Supplementary Fig. S6). However, several differences were also observed. For example, deletion was observed in partial regions on Chr7 and Chr18 on the VSMup_r2.0 against 12X, whereas duplication was observed in partial regions on Chr15 and Chr16. While the largest deletion was observed on Chr7 of VSMup_r2.0 (*V. labruscana* × *V. vinifera)* against the 12X genome (*V. vinifera*), no large sequence differences were observed on Chr7 between VSMup_r2.0 and Sylvestris_C1-2 (*V. vinifera* subspp. *sylvestris*). Instead, a large deletion was observed in Chr8 on VSMup_r2.0 against Sylvestris_C1-2. No large deletion or insertion was observed on VSMup_r2.0 (*V. labruscana* × *V. vinifera*) against EGFV_Vit.rip_1.0 (*V. riparia*), although the sequence direction was reversed in several sequences. However, less sequence similarity was observed on one half of Chr9. At this point, it is unclear whether the differences in sequence structure found in this analysis were due to differences in genome structure or assembly errors. However, when making comparisons at the gene-sequence level in future studies, the structural variations detected in this analysis should be noted.

### 3.3. Gene prediction and annotation

A total of 2.6 Gb of Iso-Seq sequences were obtained from young leaves, tendrils, flower clusters before flowering, and the skins, flesh, and seeds of mature berries at harvest (Supplementary Table S1). The sequences were then clustered and collapsed, and a total of 13,913 confident-complete (cc) gene sequences, which included ORF regions, were identified with a total length of 17.1 Mb. Then, *de novo* gene prediction was performed on the VSMuph_r2.0 genome sequences by using BRAKER2. As a result, 78,076 candidate genes were predicted on the genome, with a total length of 89.6 Mb.

The 78,076 gene sequences were then merged with the 13,913 cc gene sequences predicted by ANGEL using Iso-Seq data. The resultant 75,159 sequences with a total length of 84.1 Mb were designated VSMuph_r2.0.1. The 75,159 sequences were then classified as HC (high-confidence), LC (low-confidence), or TE (transposon elements) based on the evidence level. The numbers of predicted gene sequences classified as HC, LC, and TE were 32,827, 22,705, and 19,627, respectively (Table 1, Supplementary Table S8). The percentage of complete BUSCOs in HC was 88.9%, while those in LC and TE were 0.1% and 2.5%, respectively, suggesting that most of the protein-coding gene sequences were classified with HC.

Gene density on the VSMuph_r2.0 sequences was investigated by calculating gene sequence coverage on the genome in a 1Mb sliding window (Supplementary Fig. S7). The gene sequence cover ratio on the 19 chromosomes was 0.297 on average, with a range from 0.253 (Chr15) to 0.362 (Chr08). In most of the chromosomes, the gene sequence cover ratio was lower in the middle of chromosomes, which might represent centromere regions.

Functional gene annotation was performed by ZenAnnotation, and 25,904 of the 32,867 HC and 4,149 of the 22,075 LC gene sequences were annotated (Supplementary Tables S9 and S10). The species most frequently listed as top hit species against the ‘Shine Muscat’ genes were *V. vinifera* (84.8% in HC), followed by *V. riparia* (11.5% in HC). Among the 25,904 annotated HC genes, 18,137 genes were annotated with GO, 18,186 with GOSLIM-PIR terms, 22,151 with PFAM and 21,134 with InterPro (Supplementary Tables S11–S14).

### 3.4. Comparison with other *Vitis* genomes at the gene level

**Figure 1:**
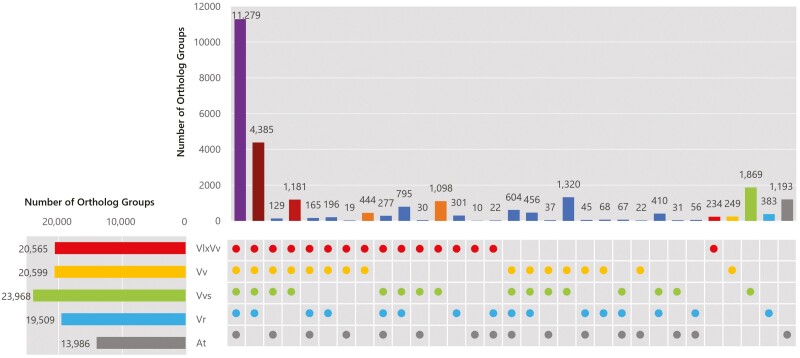
Number of shared ortholog groups of ‘Shine Muscat’ (VlxVv: *V. labruscana × V. vinifera*), *V. vinifera* (Vv), *V. vinifera* subspp. *sylvestris* (Vvs), *V. riparia* (Vr) and *A. thaliana* (At).

The translated protein sequences in the ‘Shine Muscat’ (VlxVv: *V. labruscana* × *V. vinifera*, VSMuph_r2.0.1, HC) genes were clustered and compared with the protein sequences in other *Vitis* genomes (Vv: *V. vinifera* 12X; Vvs: *V. vinifera* subspp. *sylvestris* C1-2; Vr: *V. Riparia*, EGFV_Vit.rip_1.0) and *A. thaliana* (At; Araport 11) at the amino acid level by OrthoFinder (Fig. 1). Of the 32,827 ‘Shine Muscat’ genes, 30,607 were classified into 20,565 ortholog groups. The 11,279 ortholog clusters were created with the genes commonly observed in the four *Vitis* species and *A. thaliana*. This cluster was thought to contain genes that were essential for plant survival, such as housekeeping genes. The 4,385 ortholog groups consisted of the genes commonly observed in the four *Vitis* species only, while the 1,181 ortholog groups were consisted of genes that were common to the three *Vitis* genomes, which having *V. vinifera* genomes (VlxVv, Vv and Vvs). The number of clusters that were created with the genes that were common between VlxVv and Vvs was greater than the number of clusters that were created with the genes that were common between VlxVv and Vv: i.e., 1,098 versus 444. This result suggested the possibility that the ‘Shine Muscat’ genome was more similar to Vvs than to Vv. Meanwhile, the number of ortholog groups created with the Vvs genes was 23,968, which was higher than the numbers in the other species. In addition, the number of genome-specific ortholog groups was largest in Vvs. Indeed, the number of such groups in Vvs was even larger than that in At, which was the only one of the five analyzed species that did not belong to the genus *Vitis*. Thus the gene sequence variety identified on the Vvs genome was larger than those in the five compared species, and this variety affected a larger number of ortholog groups common to VlxVv and Vvs than common to VlxVx and Vv. A phylogenetic tree of 956 common single copy genes of the four *Vitis* species and *A. thaliana* suggested that the genetic distances between ‘Shine Muscat’ and *V. vinifera* and between ‘Shine Muscat’ and *V. vinifera* ssp. *sylvestris* were almost the same (Supplementary Fig. S8).

### 3.5. Copy number variation of Akistu-21 and Hakunan against the unphased ‘Shine Muscat’ genome

**Figure 2.**
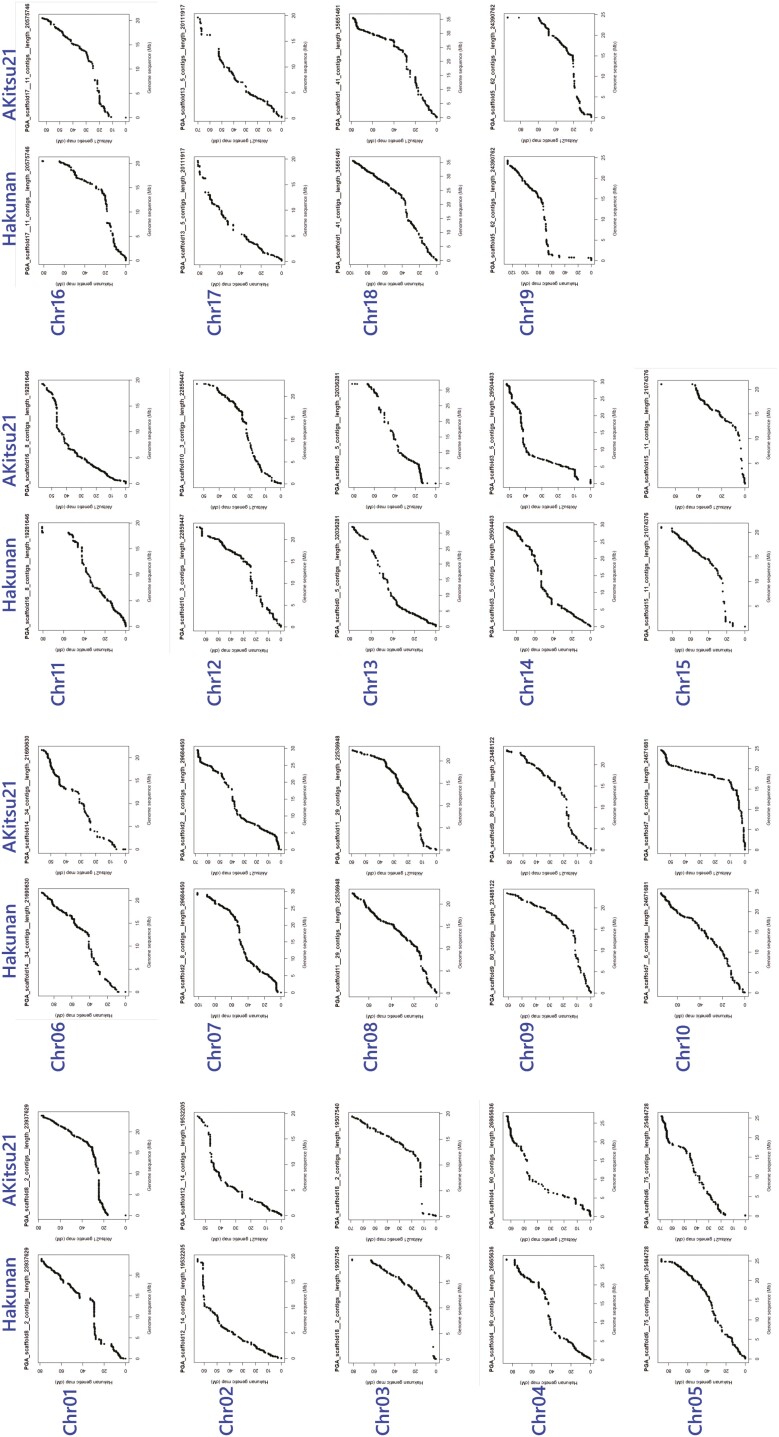
Comparisons of variant positions mapped on the longest 19 Hi-C scaffolds and the parental-specific maps. The names under the heading ‘PGA_scaffold’ represent the names of the 19 Hi-C scaffolds. The chromosome numbers given later in the analysis are shown in blue.

Following the great success of ‘Shine Muscat’ as a leading cultivar in Japan, ‘Shine Muscat’ has been commonly used as a parent in grapevine breeding in Japan. ‘Shine Muscat’ was selected from an Akitsu-21 × ‘Hakunan’ F1 population consisting of 115 individuals.^30^ In breeding, it is generally considered that the larger the population, the greater the possibility of selecting individuals with superior traits. If the breeders were to use a larger population with the same cross as used in ‘Shine Muscat’ breeding, it would be possible to create cultivars with superior traits that were not inherited by ‘Shine Muscat’. We therefore considered that knowing the genome structure variance between the two parental varieties was important in grapevine breeding, and we performed a CNV analysis against VSMuph_r2.0. CNVs showing a plus log_2_ ratio were distributed across the entire genomes in both parents. CNVs showing distributions of minus log_2_ ratio were distributed within particular regions in each parental genome, and suggested the differences of genome structures between Akitsu-21 and ‘Hakunan’ (Supplementary Fig. S9). For example, CNVs with a minus log_2_ ratio were more frequently observed in ‘Hakunan’ on 10–19Mb in Chr3, while they were more frequently observed in Akitsu-21 on 20–30 Mb in Chr13, 12–14 Mb in Chr14, and 12–30 Mb in Chr18. Of the four regions, three regions (Chr3, Chr14 and Chr18) showed low gene density, suggesting that these regions may contain centromere regions (Supplementary Fig. S7). A 20–30 Mb segment of Chr13 did not show a lower gene sequence cover ratio, suggesting that this region would contain causal variants that contributed superior agronomic traits to ‘Shine Muscat’ (Fig. 2).

## 4. Conclusion

Here, we reported the first draft genome sequence of an interspecific hybrid table grape, *V. labruscana* × *V. vinifera*. *V. vinifera* was one of the earliest plants to be sequenced, and due to technical limitations at that time, the initial genome sequencing was focused on inbred diploid lines (*V. vinifera* PN40024).^14,16^ Subsequent advances in sequencing technologies allowed in-depth analysis of heterozygous genomes in some wine grape cultivars.^17–21^ However, the genome sequences for table grapes, which are often derived from interspecific hybrids, have not been available to date. In this study, Denovo MAGIC3 was used for the assembly in order to create phased and unphased genome sequences, and successfully sequenced the genome of an interspecific table grape hybrid. Potential structural and gene sequence differences were identified between ‘Shine Muscat’ and another genus, *Vitis* accessions. Alongside a pan-genome approach,^22^ the ‘Shine Muscat’ sequence data will enhance our understanding of the composition and structure of the ‘Shine Muscat’ progenitor sequences.

The genome and gene resources created in this study may allow future identification of genes or genetic loci that contribute to the phenotypic differences within table grape varieties or between table grapes and wine grapes. Thus, the availability of the whole-genome sequence of the interspecific hybrid *V. labruscana* × *V. vinifera* will considerably facilitate future molecular genomic and genetic research in this area. The ‘Shine Muscat’ genome will act as a platform upon which future genetic studies can build, allowing the selection of favorable traits from interspecific hybrid grapes and contributing to the future development of novel interspecific hybrid varieties.

## Supplementary Material

dsac040_suppl_Supplementary_Figures

dsac040_suppl_Supplementary_Tables

## Data Availability

The sequence reads are available from the DNA Data Bank of Japan (DDBJ) Sequence Read Archive (DRA) under the Bio Project number PRJDB8610. The assembled scaffold sequences, gene sequences and annotation files are available at Plant GARDEN (https://plantgarden.jp/ja/list/t2599122).
