# Supplementary material for: De novo whole-genome assembly in an interspecific hybrid table grape, ‘Shine Muscat’
Source: DNA Res. 2022 Nov 7;29(6):dsac040. doi: 10.1093/dnares/dsac040 (PMC9724765; doi:10.1093/dnares/dsac040)

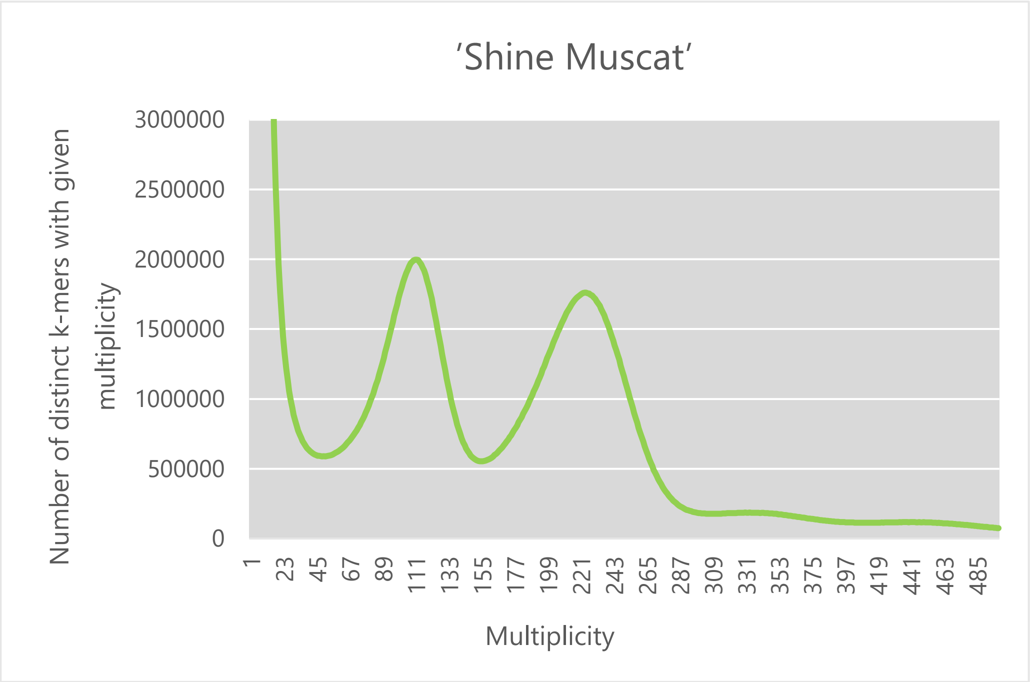

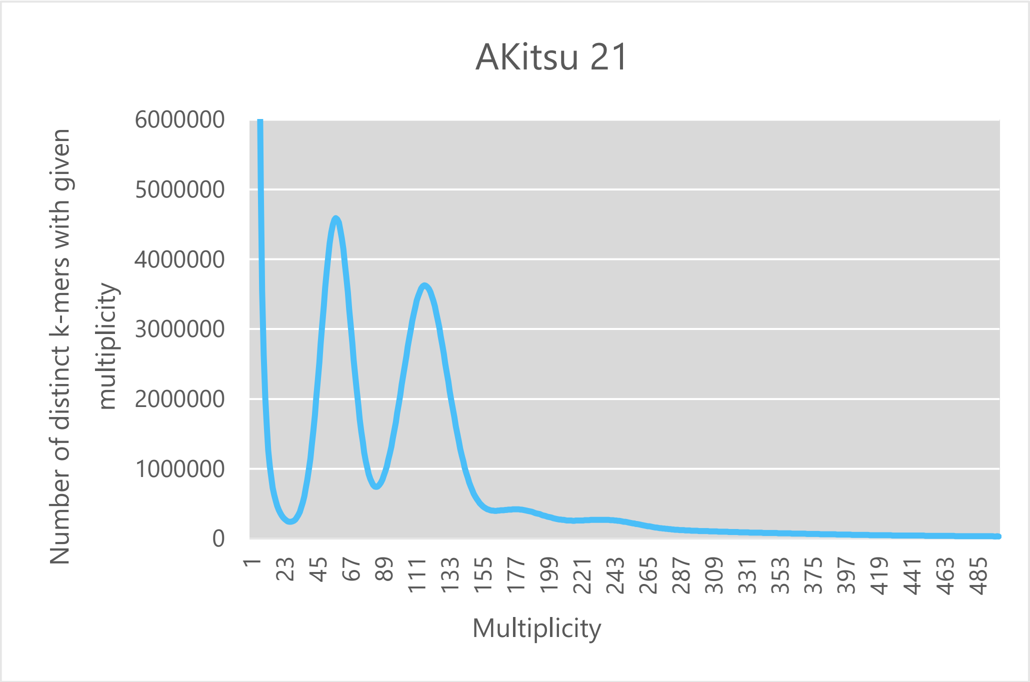

	Peak	Estimated Coverage:	Estimated Genome Size:
Shine_Muscat	111	119.8	999,223,129.4
	225	242.8	492,950,077.2
Akitsu21	58	64.9	1,112,728,631.9
	117	130.9	551,609,065.4
Hakunan	39	43.6	1,116,387,055.0
	79	88.4	551,127,786.8

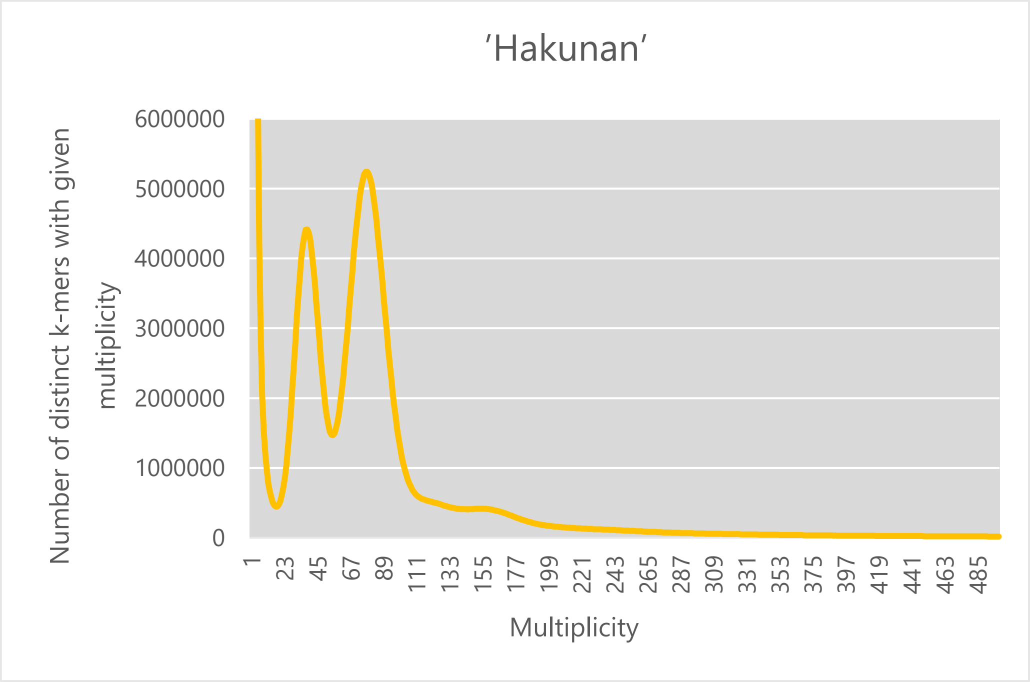

Supplementary Fig. S1. Genome size estimation using Jellyfish with the distribution of the number of distinct kmers (kmer = 17) with the given multiplicity values.

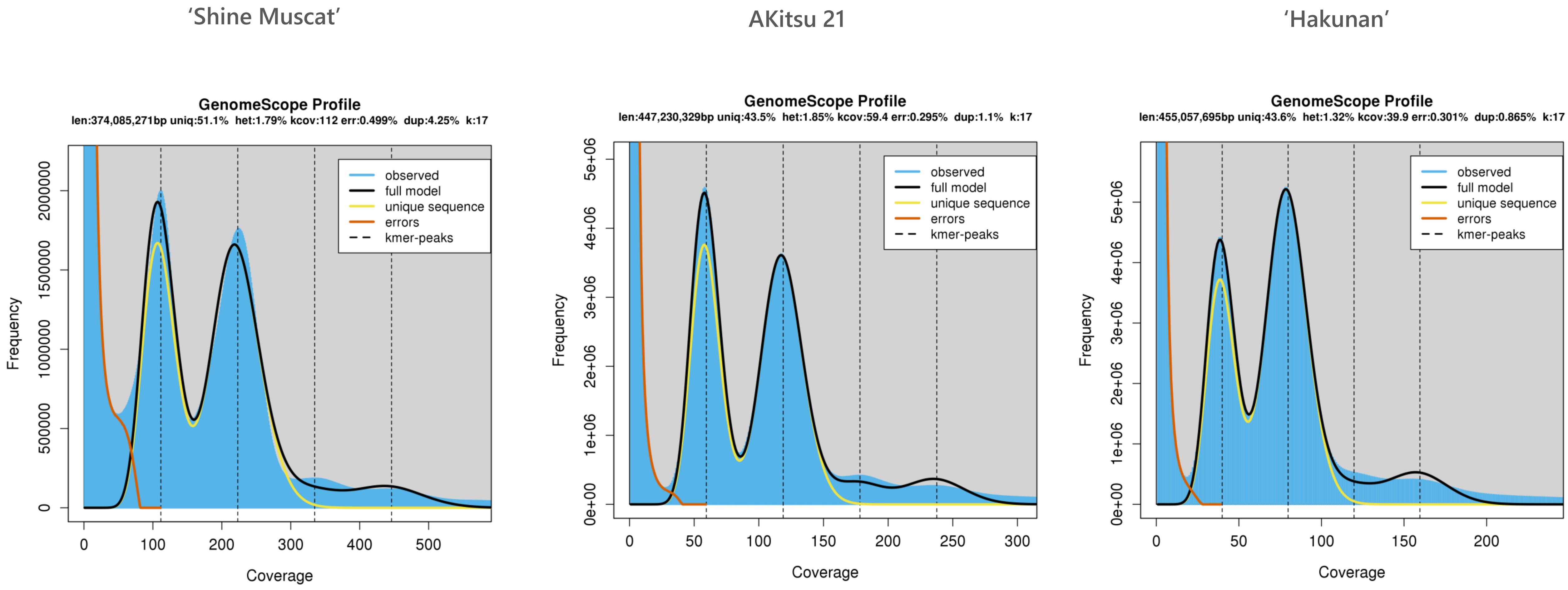

Supplementary Fig. S2. Genome size and heterozygousity estimation using Genomescope 1.0 with the distribution of the number of distinct kmers (kmer = 17) with the given multiplicity values.

Supplementary Fig. S3.

Comparisons of variant positions mapped on the longest 19 Hi-C scaffolds and the integrated linkage map.

The names under the heading ‘PGA_scaffold’ represents the names of the 19 Hi-C scaffolds. The chromosome numbers given later on the analysis were shown with blue letters.

A) Comparison with the integrated linkage map

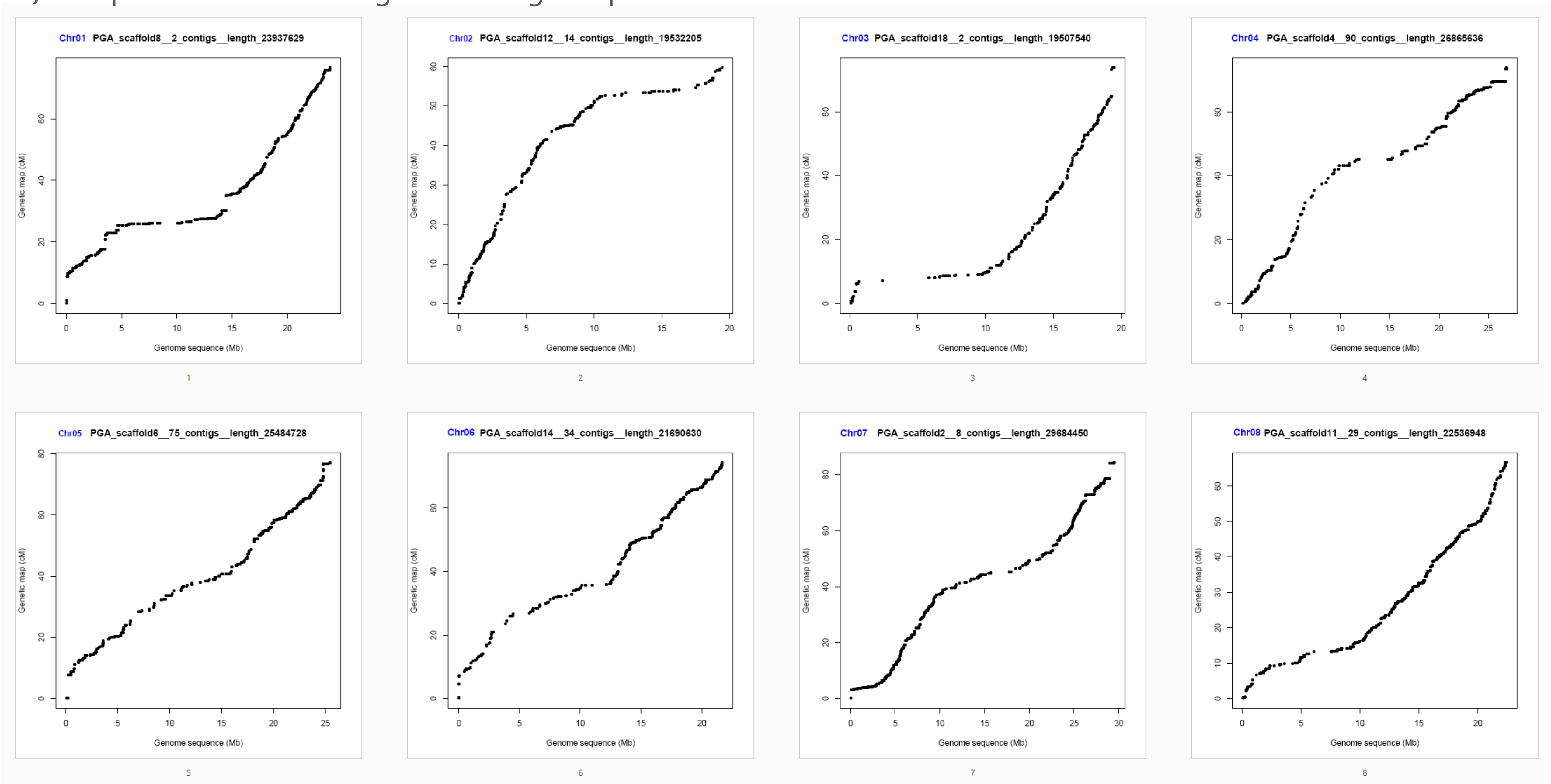

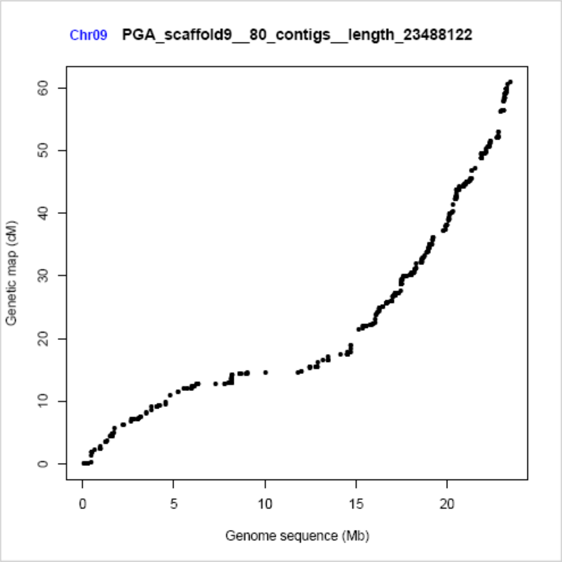

9

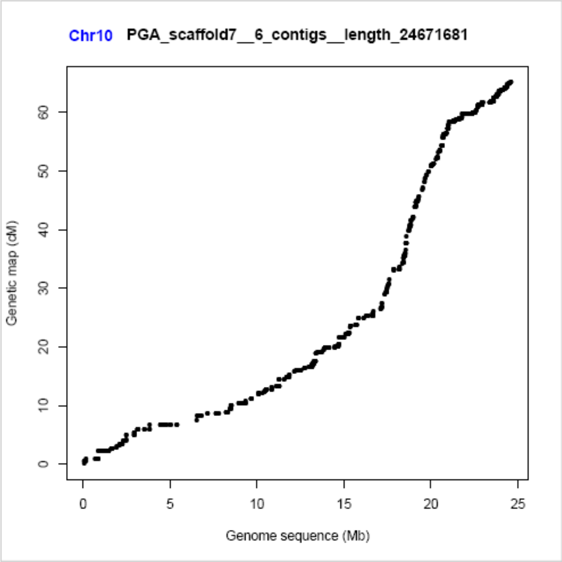

10

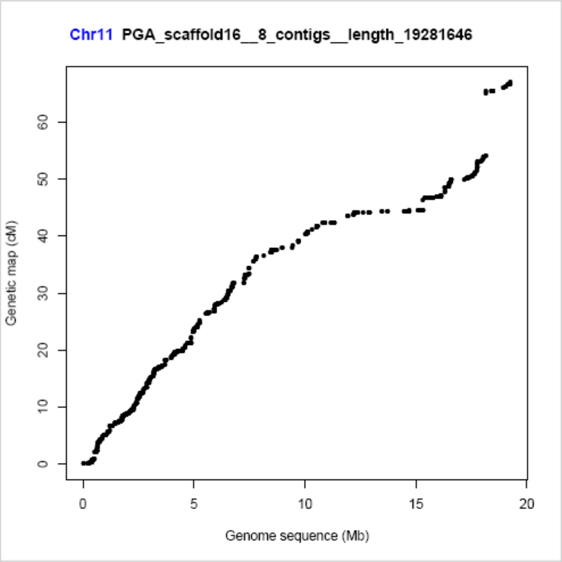

11

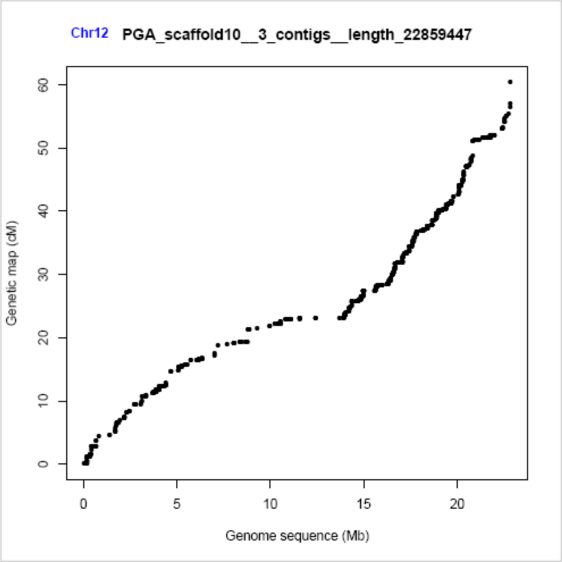

12

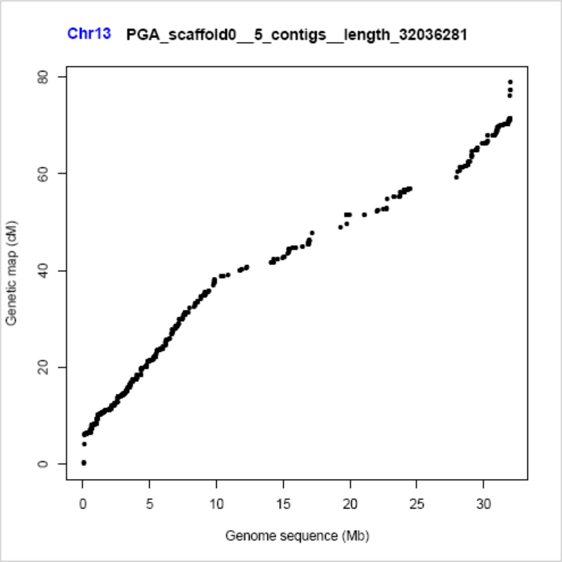

13

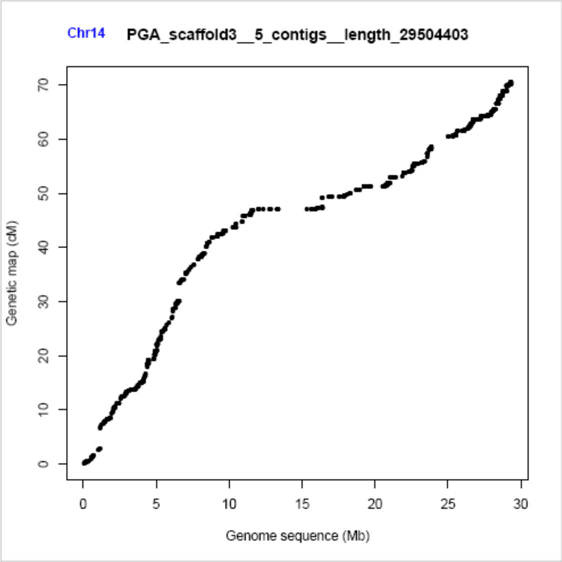

14

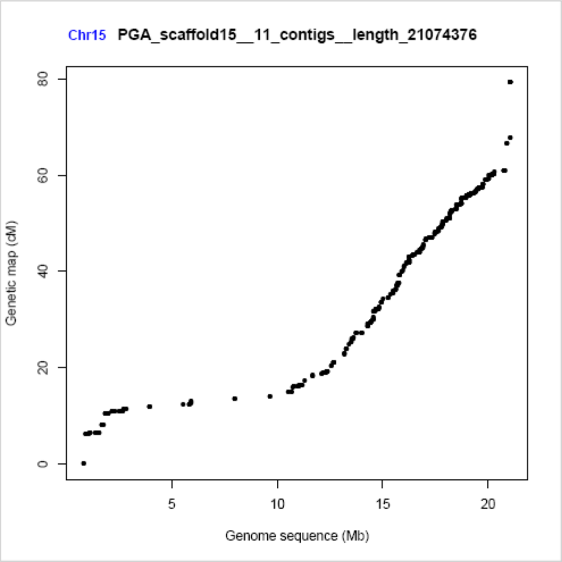

15

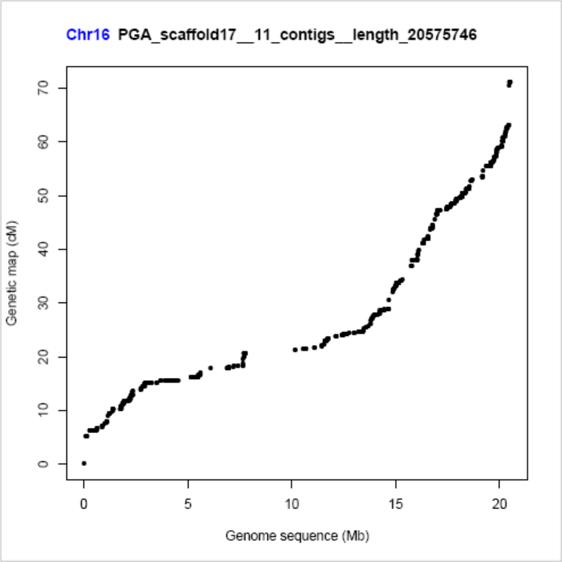

16

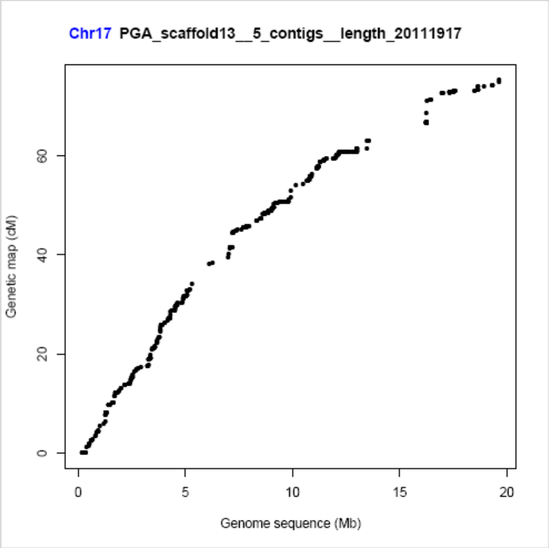
Chr17 PGA_scaffold13__5_contigs__length_20111917
Genetic map (cM)
Genome sequence (Mb)
17

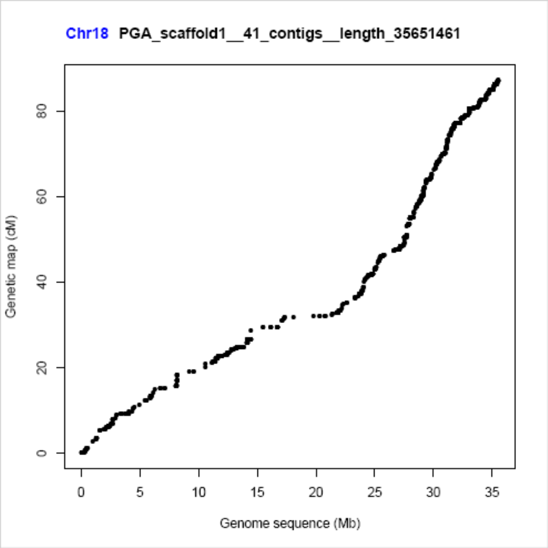
Chr18 PGA_scaffold1__41_contigs__length_35651461
Genetic map (cM)
Genome sequence (Mb)
18

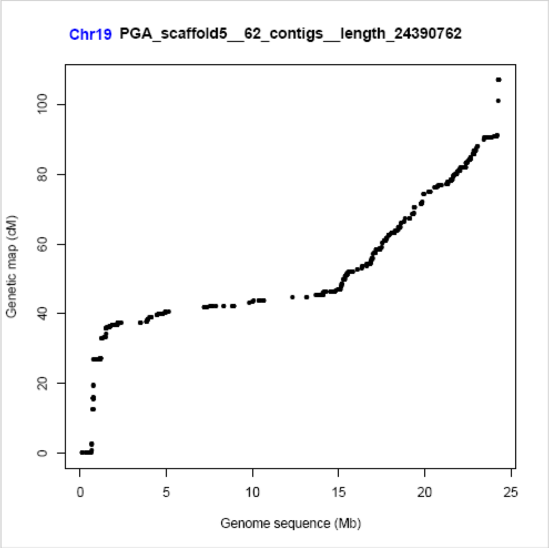
Chr19 PGA_scaffold5__62_contigs__length_24390762
Genetic map (cM)
Genome sequence (Mb)
19

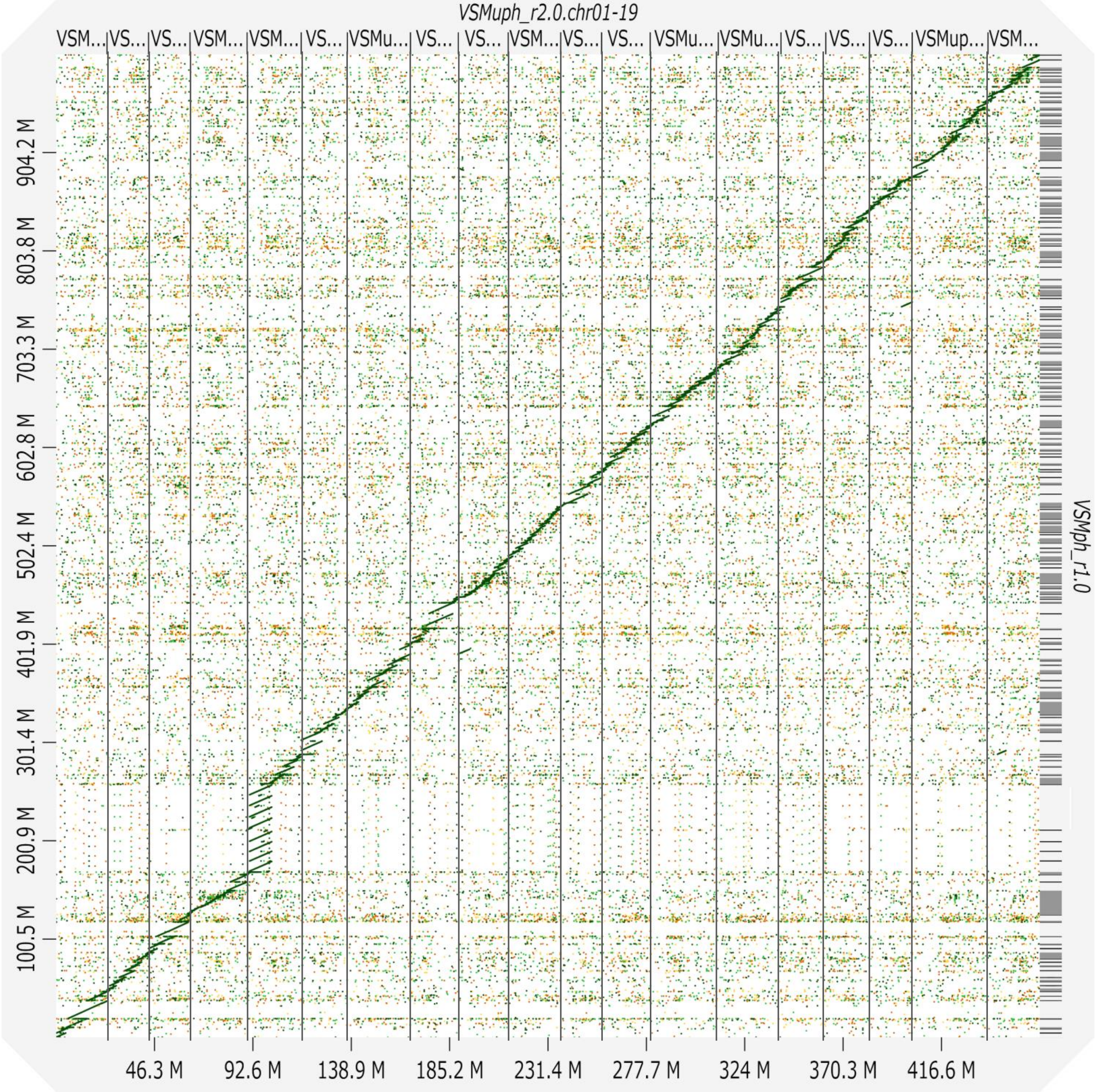

Supplementary Fig. S4. The genome sequences comparisons between the 19 pseudomolecules in VSMuph_r2.0 (unphased) and VSMph_r1.0 (phased) sequences.

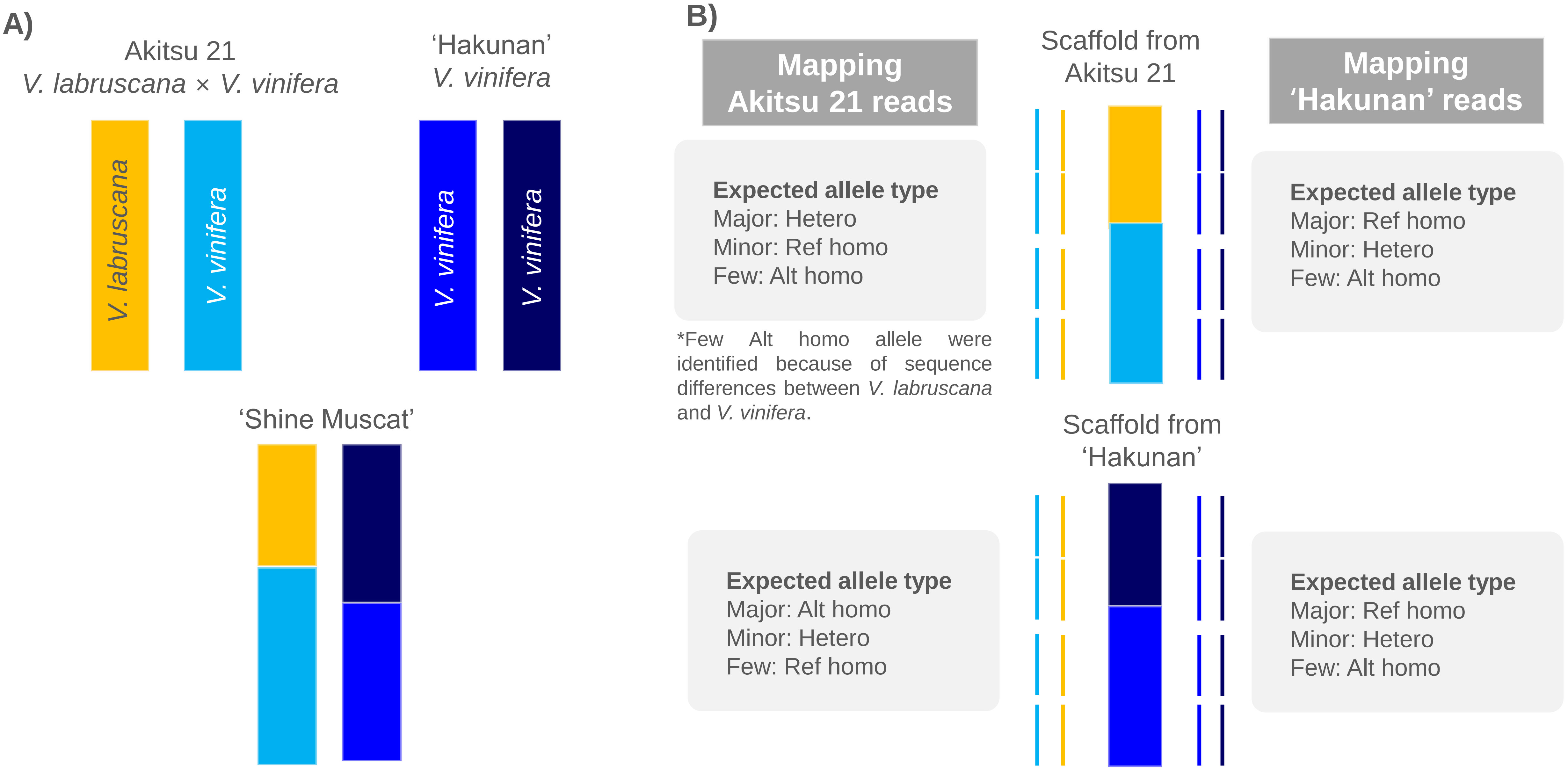

Supplementary Fig. S5. A schematic diagram of parental rads mapped onto the 'Shine Muscat' unphased genome and expected allele types on the variants. A) The haploid genome structure of the 'Shine Muscat' and the parental genomes. B) Expected allele types of Akitsu 21 and 'Hakunan' identified on the unphased 'Shine Muscat' genome sequences.

Supplementary Fig. S6. The genome sequences comparisons between the 19 pseudomolecules in VSMuph_r2.0 and those in *V. vinifera*, *V. vinifera* subspp *sylvestrie* and *V. riparia*. Blue, green and black circles show genome regions observed possible deletion, duplication and inversion on VSMuph_r2.0 against the compared sequences.

A) *V. vinifera* (12X) vs *V. labruscana* × *V. vinifera* (VSMuph_r2.0)
B) *V. vinifera* subspp. *sylvestrie* (Sylvestris_C1-2) vs *V. labruscana* × *V. vinifera* (VSMuph_r2.0)
C) *V. riparia* (EGFV_Vit.rip_1.0) vs *V. labruscana* × *V. vinifera* (VSMuph_r2.0)

A) *V. vinifera* (12X) vs *V. labruscana* × *V. vinifera* (VSMuph_r2.0)

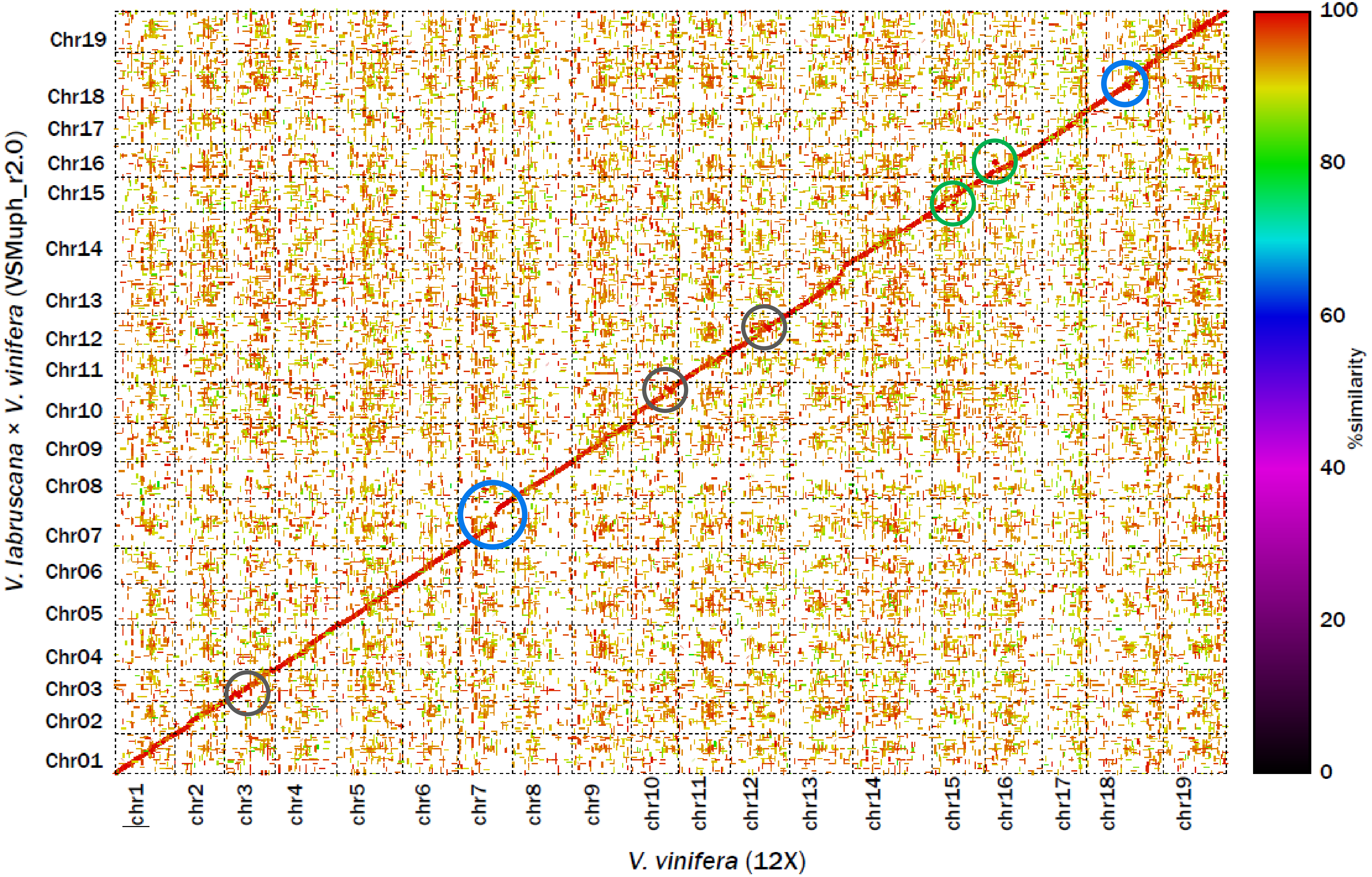

B) *V. vinifera* subspp. *sylvestrie* (Sylvestris_C1-2) vs *V. labruscana* × *V. vinifera* (VSMuph_r2.0)

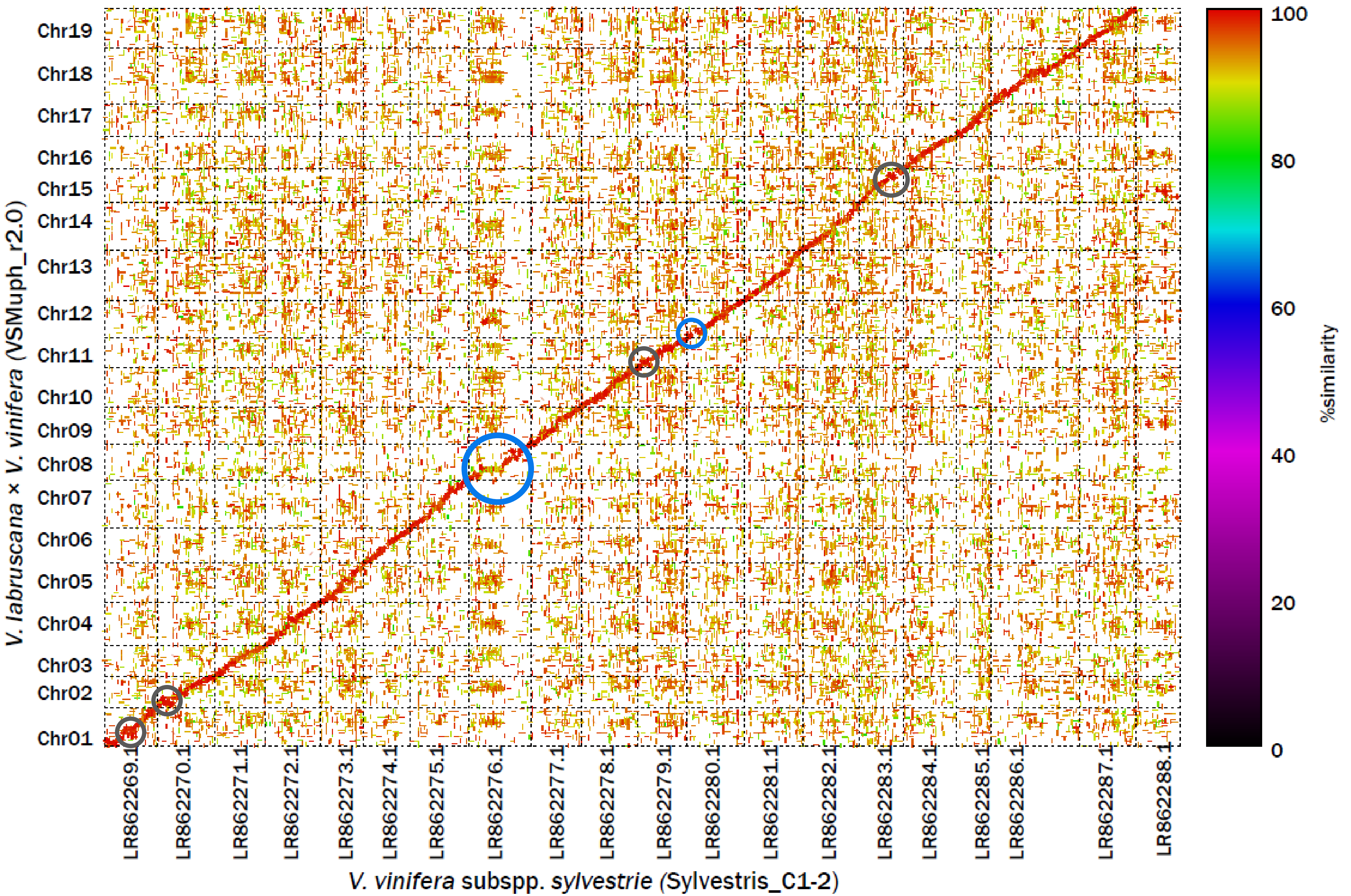

C) *V. riparia* (EGFV_Vit.rip_1.0) vs *V. labruscana* × *V. vinifera* (VSMuph_r2.0)

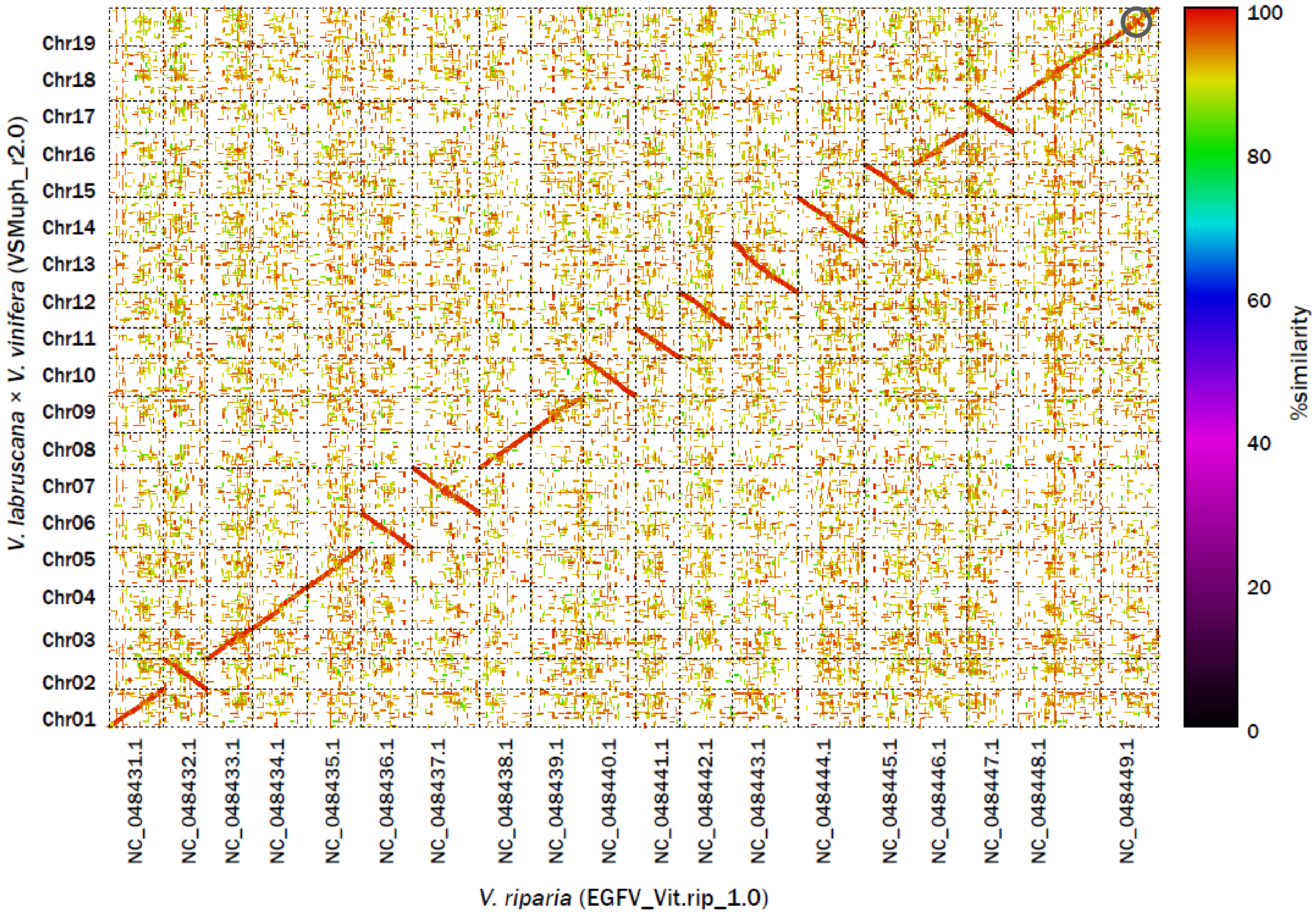

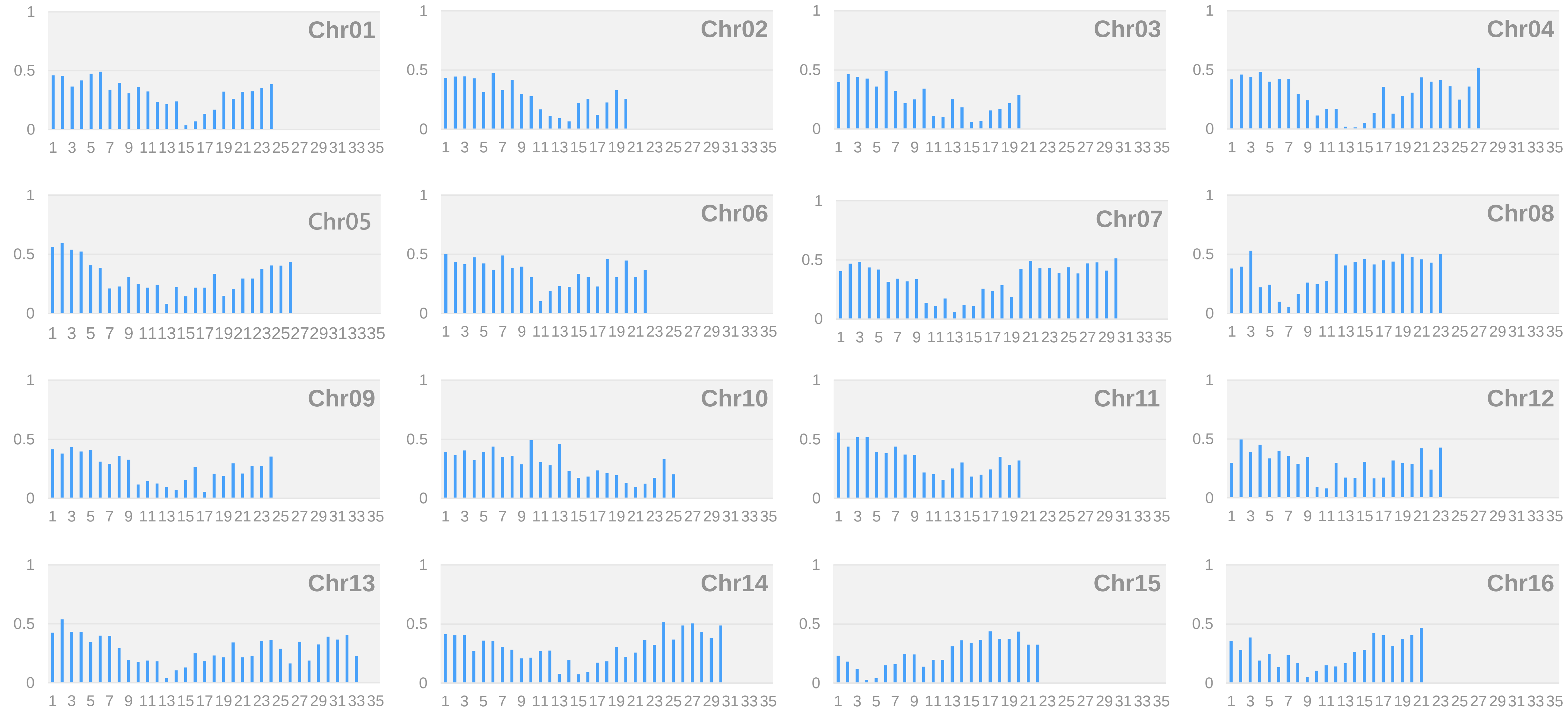

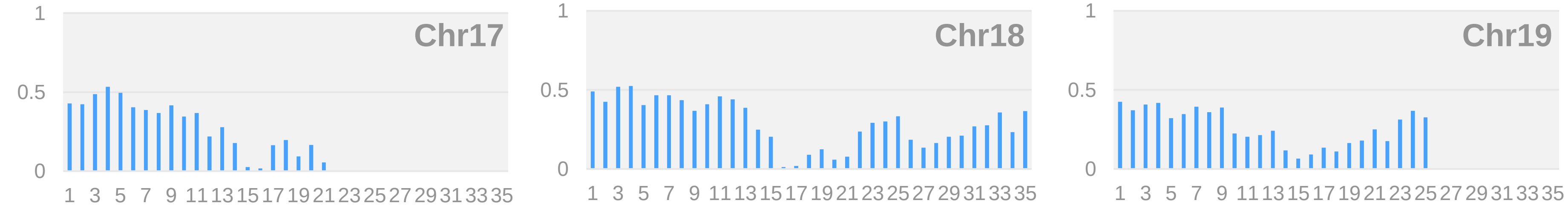

Supplementary Fig. S7. Ratios of gene sequences on the VSMuph_r2.0. X axis: 1Mb sliding windows on a chromosome. Y axis: rations of gene sequences.

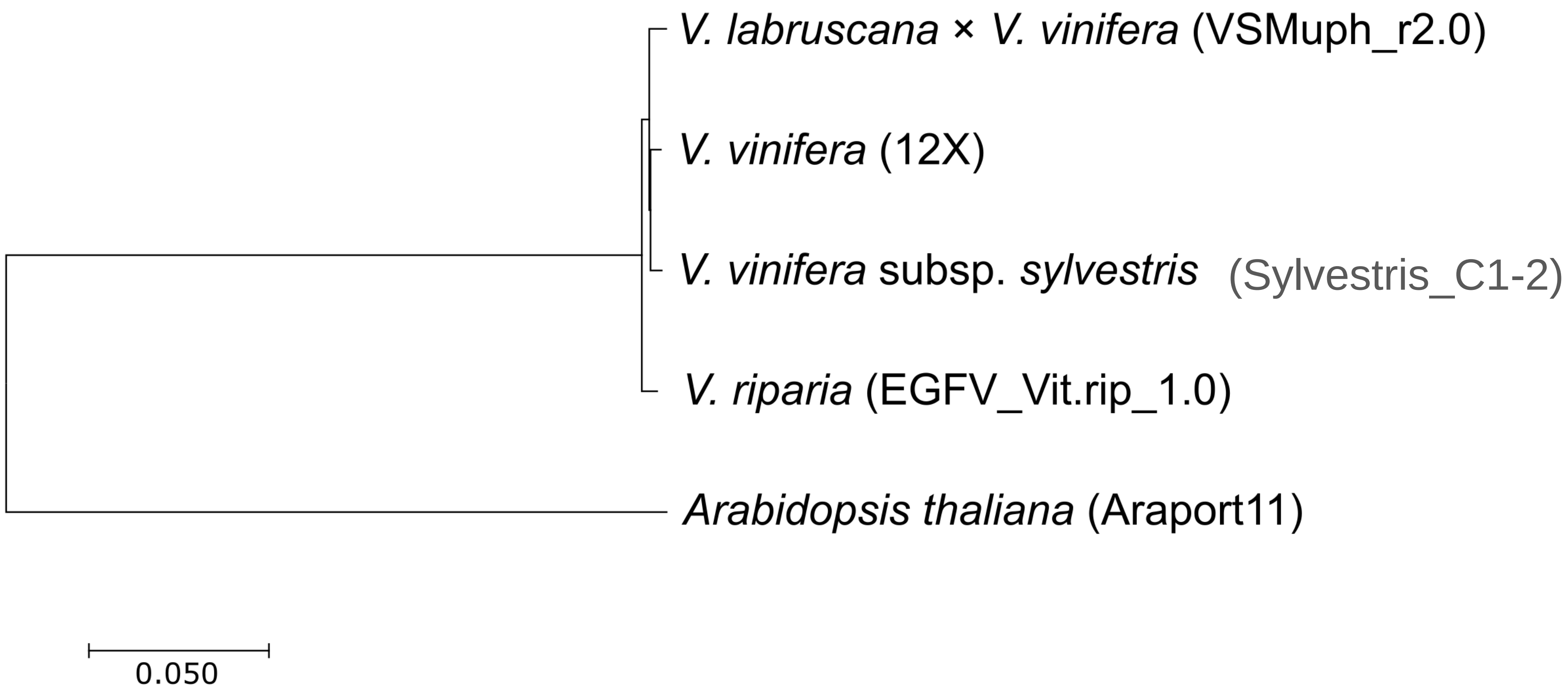

Supplementary Fig. S8. A phylogenetic tree of 956 common single copy genes of the four *Vitis* species *A. thaliana*.

Supplementary Fig. S9. Copy Number Variation of Akitsu 21 and 'Hakunan' against the unphased 'Shine Muscat' genome, VSMuph_r2.0.
The Log_2 ratio and p values was estimated by CNV-Seq software, based on mapped read count in each 1Mb sliding window.

Chr01-Chr10

Akitsu 21

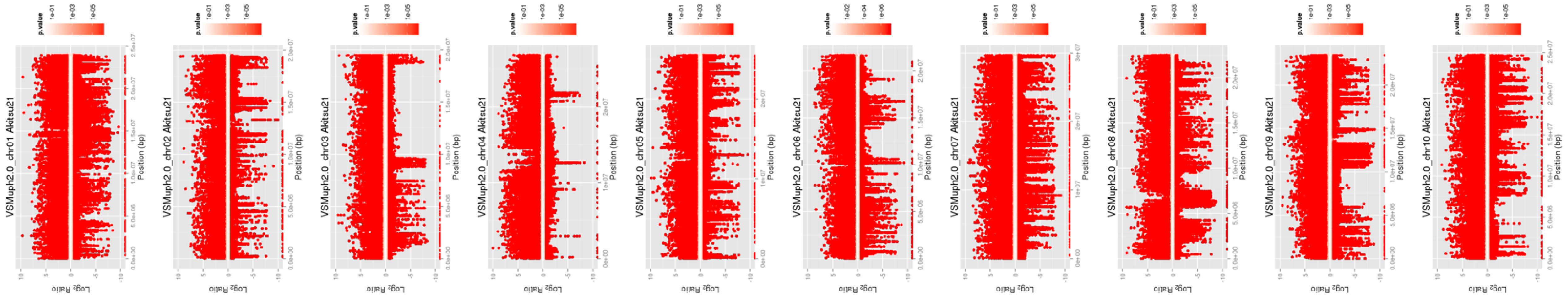

Hakunan

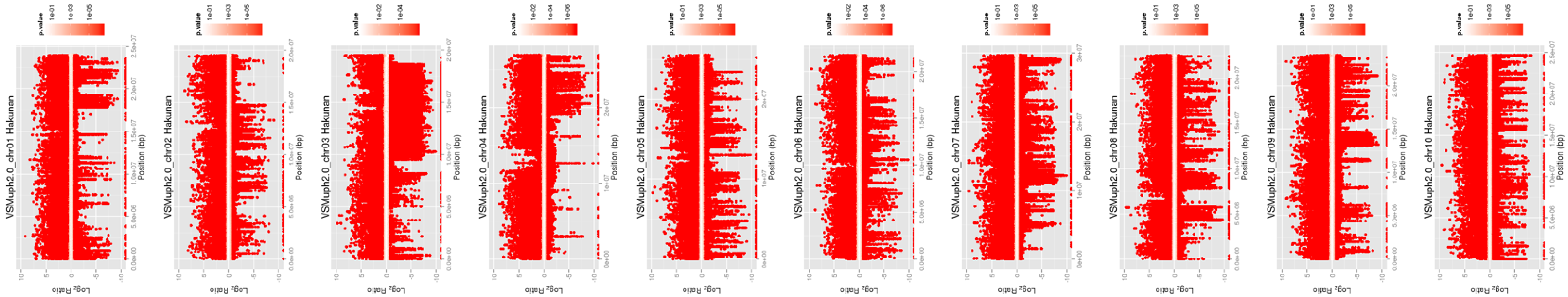

Chr11-Chr19

Akitsu 21

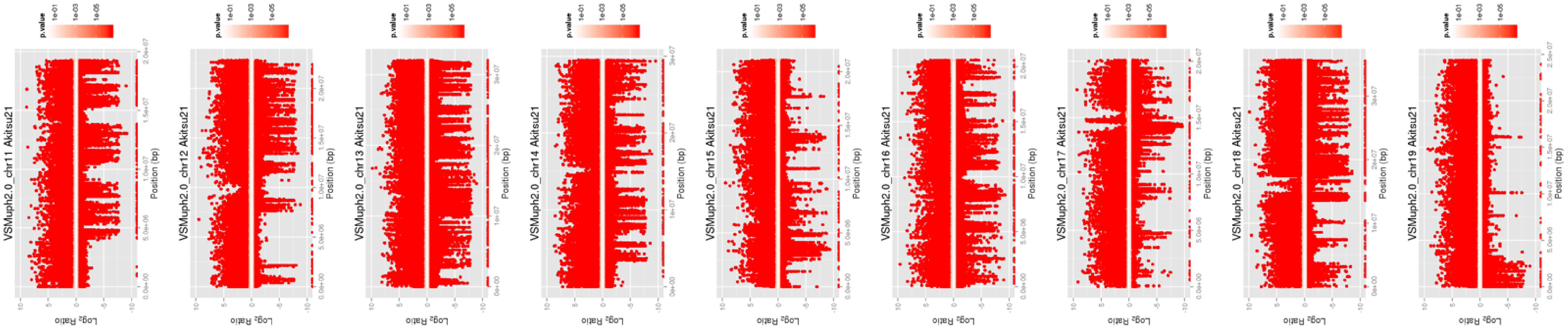

Hakunan

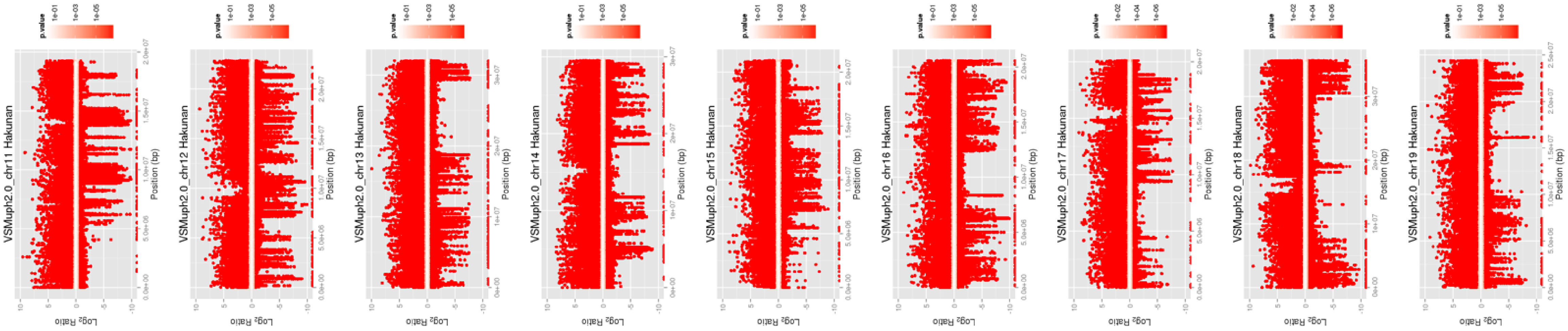

Supplement: dsac040_suppl_Supplementary_Figures [file dsac040_suppl_supplementary_figures.pdf]
